# Light Signaling in Bud Outgrowth and Branching in Plants

**DOI:** 10.3390/plants3020223

**Published:** 2014-04-23

**Authors:** Nathalie Leduc, Hanaé Roman, François Barbier, Thomas Péron, Lydie Huché-Thélier, Jérémy Lothier, Sabine Demotes-Mainard, Soulaiman Sakr

**Affiliations:** 1 Université d’Angers, L’Université Nantes Angers Le Mans, Unité Mixte de Recherche 1345 IRHS, Angers F-49000, France; E-Mails: hanae.roman@etud.univ-angers.fr (H.R.); jeremy.lothier@univ-angers.fr (J.L.); 2 SFR 4207 Qualité et Santé du Végétal, Angers F-49000, France; E-Mails: francois.barbier@agrocampus-ouest.fr (F.B.); thomas.peron@angers.inra.fr (T.P.); lydie.thelier@angers.inra.fr (L.H.-T.); sabine.demotes@angers.inra.fr (S.D.-M.); soulaiman.sakr@agrocampus.fr (S.S.); 3 Agrocampus-Ouest, Unité Mixte de Recherche 1345 IRHS, Angers F-49000, France; 4 INRA, Unité Mixte de Recherche 1345 IRHS, Beaucouzé F-49070, France

**Keywords:** bud burst, photoperiod, photoreceptor, shade avoidance, hormone, sugar, nitrogen, meristem, apical dominance, architecture, radiation

## Abstract

Branching determines the final shape of plants, which influences adaptation, survival and the visual quality of many species. It is an intricate process that includes bud outgrowth and shoot extension, and these in turn respond to environmental cues and light conditions. Light is a powerful environmental factor that impacts multiple processes throughout plant life. The molecular basis of the perception and transduction of the light signal within buds is poorly understood and undoubtedly requires to be further unravelled. This review is based on current knowledge on bud outgrowth-related mechanisms and light-mediated regulation of many physiological processes. It provides an extensive, though not exhaustive, overview of the findings related to this field. In parallel, it points to issues to be addressed in the near future.

## 1. Introduction

Branching is a key developmental process for plants that contributes to species adaptation and survival [1] and allows plants to adapt to available resources [2,3]. In crop species, it contributes to yield, either directly through production of flower-, fruit- or seed-bearing branches, or indirectly by increasing the competitive ability of crops against weeds [4,5] or by modulating the dynamics of pest infestation [6]. It also modulates plant shape and compactness, which influence plant visual quality in ornamental plants [7].

Branching results from several interrelated processes: axillary bud formation, dormancy induction and release, bud outgrowth (involving growth of preformed leaves, internode extension, and initiation of new leaf primordia by the shoot apical meristem (SAM)), and then shoot extension. Environmental factors finely control the activity-dormancy cycle of buds and shoot growth [8,9,10,11,12,13,14]. Among these, light is a major factor that significantly impacts branching in numerous species. Still, our knowledge about the molecular mechanisms from light perception by buds to bud outgrowth is scarce and fragmented. This knowledge gap contrasts with the importance of bud outgrowth in plant physiology and with the high responsiveness of bud outgrowth to light. This review is an overview of the regulatory molecular network of light signaling in bud outgrowth and proposes relevant topics for future investigations. 

## 2. Plant Branching and Bud Outgrowth Are Regulated by Light Intensity and Quality and by the Photoperiod

### 2.1. Impact of Light Intensity

In many species, bud outgrowth is modulated by light intensity. In herbaceous species such as the monocots *Lolium perenne* [15] or *Triticum aestivum* [16], low-intensity light caused decreased tillering, while high-intensity light stimulated branching in several shrubs and trees such as *Vaccinium bracteatum* [17,18], *Abies balsamea*, *Picea abies*, *Pinus sylvestris* [19,20], *Litsea acuminata* [21], *Fraxinus pennsylvanica* [22], *Rosa hybrida and R. wichurana* [23]. An absolute requirement for light was even observed for basal bud outgrowth in rose [23,24] and in pea [25]. In rose, darkness fully inhibited SAM organogenetic activity and the expansion of preformed leaves required for outgrowth. Yet, as low a Photosynthetic Photon Flux Density (PPFD) as 2 µmol∙m^−2^∙s^−1^ was sufficient to trigger outgrowth [23]. 

Light intensity not only interferes with the potential of individual buds to grow out but also, both spatially and temporally, on the bud outgrowth gradient along a shoot. For example, in *R. hybrida* “Radrazz” and *R. wichurana*, when dark conditions were applied to the upper part of the stem, outgrowth of distal buds was inhibited, and this allowed for otherwise quiescent median and basal buds to sprout [26]. In *R. hybrida* “Radrazz”, bud outgrowth in the median zone of the primary axis was increased when cuttings were grown initially under low-intensity light (91 µmol∙m^−2^∙s^−1^) until the floral bud was visible and then exposed to high-intensity light (580 µmol∙m^−2^∙s^−1^), in comparison to constant exposure to high-light intensity (580 µmol∙m^−2^∙s^−1^) [10]. 

### 2.2. Impact of Light Quality

The spectral quality of light is an important source of information for plants about their environment. Perception of light quality allows plants to sense the presence of other plants in their neighborhood, as well as shade, daytime and seasons. These cues help them adjust their development to better compete for resources, detect changes in daylength [27,28,29,30] or, on a longer term basis, prepare for drastic environmental changes such as low temperatures in winter. All of these signals can modulate the capacity of buds to grow out.

A well-known case is the developmental response of plants to shade conditions: under shade, *e.g*. in dense culture conditions or under the tree canopy, plants compete for light and develop a growth strategy called the shade-avoidance syndrome (SAS). SAS is more particularly characterized by a reduced capacity for axillary buds to grow out. As a result, plants can allocate resources so as to promote main shoot growth and leaf orientation towards sunlight and early flowering. In turn, early flowering can also influence branching, in particular in indeterminate species [31]. SAS is triggered when plants sense a low ratio of Red (R) to Far-red (FR) lights (R:FR) and reduced blue light intensity in their surroundings [32,33,34]. When red and blue wavelengths are absorbed by neighbor plants for photosynthetic assimilation and photomorphogenesis, and the reflection of FR radiation by their green tissues decreases R:FR and blue light intensity in the full light spectrum, each plant senses the presence of competing neighbors before actual shading [35,36]. 

Reduced shoot branching due to low R:FR has been reported in many crop plants such as *Lolium multiflorum*, *Paspalum dilatatum* [37,38], *Hordeum vulgare* [39], *Eragrostis curvula* [40], *Trifolium repens* [41], *Brassica juncea* [35]. In ornamentals too, investigations have aimed to understand how light quality can modulate branching. In *Disanthus cercidifolius*, *Crateagus oxyacantha*, different *Rhododendron* [42] and *Rosa* cultivars [23,43], R light promoted bud outgrowth while FR light was highly inhibitory in *Lilium* [35] and in *Rosa* [23,44,45].

Fewer investigations have been carried out so far on the impact of blue light on bud outgrowth, and results sometimes differ depending on species: blue light stimulated bud outgrowth in *Triticum aestivum* [44], *Prunus cerasifera* [46], and *Rosa* [23,47] whereas it reduced it in *Solanum tuberosum* [48]. Even within a single species, plants’ response to blue light can differ among varieties, as shown in tomato [49].

### 2.3. Impact of the Photoperiod

Photoperiodic control is a well-known environmental factor that induces bud formation and dormancy in many perennial species (for a review see [13]). However, less is known about the effect of daylength on the capacity for buds to grow out. In the ornamental plant *Rhododendron catawbiense*, the shoot branching pattern was modified by the photoperiod [50]: the distal shoots of single shoot plants developed under long days (LD) (16 h day) while basal shoots remained quiescent. However, under short days (SD) (8 h day), the growth potential of distal buds strongly decreased. This modified shoot branching pattern in turn reduces apical dominance, whereby the growing apex keeps buds located down the same axis dormant (see [51] for a review). In non-horticultural plants such as *Arabidopsis thaliana*, increasing the frequency of the light/dark transition (7 h/7 h) strongly inhibited the development of lateral shoots from the axillary buds of rosette leaves [52,53]. Yet, in pea, short photoperiods (8 h day) increased the formation of basal branches [54]. The photoperiod also controls the induction of flowering in photoperiod-sensitive plants. Therefore the growth response of vegetative buds to the photoperiod may—at least partly—result from altered flowering. This should be taken into account when analyzing the potential for vegetative bud growth under photoperiodic control.

## 3. Photoreceptors Involved in the Control of Branching

Few data are presently available on the individual role of each plant photoreceptor in the control of bud outgrowth. Results have been obtained only in a limited number of plant species: *Arabidopsis*, pea, tomato and sorghum, in which photoreceptor mutants have been characterized. 

### 3.1. Phytochromes

Phytochrome B (PHYB) is involved in the response of bud outgrowth to light in *Arabidopsis* [55,56] and sorghum [57,58]. In these species, *phyB* loss-of-function mutants exhibited a reduced branching capacity compared to the wild-type (WT), suggesting a promoting effect of PHYB on bud outgrowth in the WT background. In *Arabidopsis*, low R:FR caused the same reduced bud outgrowth as the *phyB* mutation, indicating that PHYB acts through the sensing of R:FR and that high R:FR promotes bud outgrowth by PHYB [55]. In *Sorghum*, supplemental FR light in a background of white light reduced bud outgrowth in the wild type in the same way as in *phyB-1* mutants [58].

The action of PHYA is plant-dependent. In rice, *phyA* mutation did not bring about any distinguishable phenotypic characters from the WT, bud outgrowth capacity included [59]. Yet, in pea, *phyA* mutation induced an increased branched phenotype: in wild-type cultivar “Torsdag” pea plants grown under natural light and a long photoperiod, no lateral branch was produced. However, *phyA* mutation caused both basal and aerial buds to sprout and numerous branches to grow all along the main stem [60]. This latter phenotype was also observed in the WT plants grown under a SD photoperiod. Moreover, when daylength was extended to LDs using FR-rich radiation, PHYA emerged as the primary phytochrome responsible for the detection of FR-rich photoperiod extension involved in daylength detection. 

From these results, it appears that phytochromes are involved in the control of bud outgrowth through the sensing of daylength and R:FR, so that plants can get cues about established or future shade conditions. 

### 3.2. Cryptochromes

Cryptochromes are other important photoreceptors that contribute to the photocontrol of plant development through the perception of blue light. Although *cry* loss-of-function mutants have been obtained in several species (pea: [61], tomato: [62], *Arabidopsis*: [63]), no impact of *cry* mutation on bud outgrowth has been reported. However, *CRY2*-overexpression in tomato plants relieved the inhibition of axillary meristems due to apical dominance and the plants exhibited an extensively branched phenotype [64]. Interestingly, such a result was not observed in *Arabidopsis* *CRY1* or *CRY2* overexpressors [65,66,67], suggesting different developmental controls by CRY in these two plant species. *Cry1* mutation previously induced reduced elongation of axillary branches in tomato [62]. It then appears that branching is under the control of cryptochromes in tomato: while CRY2 is believed to promote bud outgrowth, CRY1 is thought to stimulate the growth of the developing axillary branches.

## 4. Site(s) of Light Perception in the Control of Bud Outgrowth

In order to understand the effect of light on bud outgrowth, it is important to determine where the site(s) of light perception involved in the control of bud outgrowth is/are. Such a question is particularly important to clarify to what extent bud outgrowth photocontrol is an autonomous process mainly regulated by light perception by the bud itself, or a non-autonomous process involving light signal transduction from other parts of the plant toward the buds. The non-autonomous process is involved in the control of the flowering fate of the meristem in photoperiod-sensitive plants: the LD light signal is perceived by the leaves and induces higher transcription levels of the *Flowering Locus T (FT)* gene and transfer of the FT protein toward the shoot apical meristem *via* phloem transport [68].

In rose, where bud outgrowth strongly depends on photocontrol, buds are perception sites—in defoliated plants at least—that can trigger bud outgrowth on their own [23]. Therefore we can wonder where exactly they perceive light, which light sensors are present and which of them are involved in the control of bud outgrowth. In plants, light is perceived through pigments that collect light for photosynthesis (mainly chlorophylls and carotenoids in higher plants) and photoreceptors that control photomorphogenetic responses. Photosynthetic pigments are found in all green tissues. A bud’s green scales, young preformed leaves, leaf primordia as well as meristems contain chlorophyll and are thus likely to have photosynthetic activity. Regarding photoreceptors, no specific study has been carried out so far to identify the types and the distribution of the photoreceptors present in buds and their component organs. At the whole-plant level, photoreceptors have a wide distribution across the plant: they are distributed among all organs, even those that grow in dark environments such as the roots, and in all tissues, even internal ones such as vascular tissues [69,70,71]. Based on this wide distribution of light sensors in various plant organs, we can expect that (i) buds will contain all photoreceptor types; and (ii) any particular qualitative distribution of photoreceptors will have a limited effect on the regulation of their response to light. Moreover, phytochrome genes are constitutively expressed in all plant organs, with only modest changes in transcript levels across developmental stages [70,72,73]. The levels of phytochrome proteins except PHYA are also quite stable throughout development in each given organ and through diurnal and circadian light cycles [74]. This suggests that, except for light-labile PHYA, mechanisms other than transcriptional and translational regulation of *PHY* genes such as photoconversion, interaction with specific partners or biochemical activity are involved in the photocontrol of bud outgrowth as in the rest of the plant [70,75]. 

## 5. Light Transduction in Bud Outgrowth and Interaction with Growth Processes

### 5.1. From Photoreceptors to the Early Steps of Light Signaling

Little is known about the first steps of the transduction of a light signal from its photoreceptor down to bud outgrowth processes. A lot of knowledge has been gathered these past years in the identification of photoreceptors’ early interacting partners during light control of seed germination [76], hypocotyl de-etiolation [77,78,79,80], the shade-avoidance syndrome of seedlings [81,82,83,84,85] or phototropism [86], but no such study was carried out on bud tissues during outgrowth. Important roles for Phytochrome-Interacting Factors PIF1,3,4,5 and 7 were demonstrated in the above studied models. Overlapping, but also specific actions of these PIFs were shown according to the biological process studied or the light condition used ([87]). Thus, the same investigations need to be undertaken for bud outgrowth in order to identify the interactions that are specific and those that are common to the previously studied light responses. Regarding the early steps of cryptochrome signal transduction, only two interacting partners of these photoreceptors have been identified [88]. The CIB1 (Cryptochrome-Interacting Basic-helix-loop helix) protein interacts with CRY2 under blue light and binds to the promoter of certain genes to activate their transcription [89], while SPA1 (Suppressor of Phytochrome A) is involved in the post-transcriptional regulation of gene expression under blue light. SPA1 is a positive regulator of the E_3_ Ubiquitin ligase Constitutive Photomorphogenesis Protein 1 (COP1), and interaction with CRY leads to the suppression of the COP1-dependent degradation of transcriptional regulators [88,90,91]. No report has dealt with the role of these proteins in the photocontrol of bud outgrowth so far.

### 5.2. Central Integrators

Investigations have been made further down the signaling pathway. For instance, in *Sorghum*, PHYB contributes to the photoregulation of *TEOSINTE BRANCHED1* (*TB1*) [58,92], a major tillering-repressor gene [93,94]. A similar study in *Arabidopsis* revealed that the functionality of two homologous genes of *TB1*, namely *BRC1* and *BRC2*, was required for the transduction of the light signal through PHYB [55]. The authors further showed that BRC1 and BRC2 contributed differently to the transduction of the light signal and suggested that these two branching integrators belonged to two distinct light signaling pathways of bud outgrowth. Moreover, when FR was added to white light to mimic shade, *BRC1* expression was promoted in *Arabidopsis* without affecting *BRC2* expression [95]. Transcriptomic profiling of *brc1* mutant buds under shade revealed groups of genes under the control of BRC1. Among them, some abscisic acid (ABA)-related genes are up-regulated while cell cycle and protein synthesis-related genes are down-regulated when BRC1 is activated [95]. This study opens the way to a better understanding of the molecular network controlled by this branching integrator under shade conditions.

### 5.3. Interaction of Light and Hormone Signaling Pathways during Bud Outgrowth

Several hormones play a key role in the control of bud outgrowth. Their potential interactions with light in this process are discussed below:

#### 5.3.1. Interaction with Cytokinins

Cytokinins (CKs) are the only hormones known to stimulate bud outgrowth. They act by decreasing the expression of *BRC1* [96,97] and by antagonizing auxin and strigolactones in apical dominance [98,99,100,101,102,103]. When apical dominance ends, CK levels increase in the stem [104,105] and in the bud as neo-synthesized CKs are transported from the stem to the bud [105]. Moreover, CKs promote auxin synthesis and export to the stem and buds, and this in turn induces bud outgrowth [99,106,107]. In buds, CKs control SAM organogenesis and elongation of the preformed organs [108,109,110,111] through a fine regulation [108,112,113,114,115,116]. 

Little is known about the interaction between CKs and the photocontrol of bud outgrowth. In pea, when etiolated epicotyls bearing cotyledon axillary buds were dipped into a sucrose solution and exposed to red light, CK application increased bud outgrowth and enhanced sucrose incorporation into the buds [117]. In tomato and *Arabidopsis*, SAM organogenesis was inhibited in the dark but the absence of light was offset by CKs [118]. These two works evidence a possible interaction of the CK- and light-signaling pathways in the control of SAM activity and bud outgrowth. However, limited information is currently available about the cross-talk between these two pathways in the control of bud outgrowth. 

#### 5.3.2. Interaction with Auxin

Auxin is a key component of apical dominance. As such, it plays a crucial role in the control of bud outgrowth. It is mainly produced by the young growing organs of the apex [119,120], it is transported toward the root through the xylem parenchyma in a polarized way mainly under the control of a class of auxin efflux facilitators, the PIN-FORMED (PIN) proteins [121,122]. Below the apex, auxin inhibits the outgrowth of the axillary buds located on the same axis, and thus controls the systemic regulation of shoot branching [51,119,123]. Auxin does not enter the bud [124,125], and acts through indirect mechanisms. On the one hand, the auxin stream inside the stem inhibits CK synthesis inside the stem [105] and upregulates the synthesis of strigolactones [126,127], a class of hormones that inhibits bud outgrowth (see below). On the other hand, the auxin stream in the stem prevents the establishment of a polarized auxin transport (PAT) between axillary buds and the stem, and this prevents buds exporting their own auxin [128,129,130]. PAT regulation in buds is crucial for the formation of leaf primordia and vasculature in active buds [131,132,133,134].

Up to now, few results have been obtained about the impact of light on auxin signaling in the control of bud outgrowth. In *Arabidopsis* buds, PHYB positively regulates the expression of *TRYPTOPHAN AMINOTRANSFERASE OF ARABIDOPSIS1* (*TAA1*), a gene that encodes a rate-limiting enzyme in auxin biosynthesis whose expression correlates with bud outgrowth [55]. In tomato meristem, light regulates the localization of PIN1 at the plasma membrane and the initiation of primodia in a CK-dependent manner [118]. In the same line, light upregulates organogenesis during bud outgrowth in rosebush axillary buds [23]. In other biological systems, an interplay between the auxin and light signaling pathways has been reported. For example, auxin accumulation in the elongating organs of seedlings during SAS was triggered by a low R:FR ratio, and mutation of *TAA1* led to a partial loss of this response [135]. During phototropic growth responses too, light-directed plant growth was mediated by asymmetric auxin distribution in tissues, and this effect was mainly due to a control of PIN polarization [136,137,138,139,140]. These observations raise the question of how light can regulate stem PAT and the establishment of a PAT between bud and stem, as these are believed to have opposite effects on bud outgrowth. 

#### 5.3.3. Interaction with Strigolactones

Strigolactones, or the ultimate signal derived from them, inhibit bud outgrowth [51,141,142]. The signaling pathway involves the rice *D14/D88/HTD2 gene* and *Petunia hybrida DAD2* gene coding for an α/β hydrolase that may act as a receptor of the strigolactone molecule [143]. Upon perception, this α/β hydrolase interacts with the F-box protein MAX2 belonging to a SCF complex to target positive branching integrators to proteolysis. MAX2 was also shown to promote expression of negative branching integrators such as BRC1 [144,145]. 

Little is currently known about the effects of light on the strigolactone pathway and their subsequent impacts on bud outgrowth. Still, the basal branching habit of strigolactone-synthesis-gene *rms1* and *rms2* pea mutants under SD shifted to more aerial branching under LD [54]. This suggests an interconnection between the photoperiod and strigolactones. Besides, MAX2 was evidenced as a positive regulator of hypocotyl de-etiolation after light exposure and its expression was induced by R and FR light, probably through PHYA and PHYB signaling [146]. The interconnection between the strigolactone- and phytochrome-signaling pathways in *Arabidopsis* shoot branching has been confirmed [55]. Loss-of-function mutants were used to show that full functionality of the *MAX2* and *MAX4* genes was required for PHYB to have both a stimulatory effect on bud initiation and outgrowth and a repressive effect on correlative inhibition between axillary branches. In sorghum, where *phyB* mutation or FR treatment induced bud dormancy and therefore bud outgrowth inhibition [58], these two inhibitory conditions were correlated with a strong increase in *SbMAX2* expression in buds [92]. Altogether, these studies suggest that the *MAX2* gene acts downstream of a light-signaling pathway that involves PHYB photoreceptors and thus contributes to the inhibition of bud outgrowth under particular light conditions. 

The relationship between the changes induced by light treatment in the branching gradient along a stem and the pattern of *MAX* gene expression along the same stem was studied in *R. hybrida* “Radrazz” [26]. The decreasing gradient of bud outgrowth from the distal part to the basal part of the parent shoot observed in rose under natural light cannot be explained by the establishment of a negative expression gradient of *RwMAX1*, a strigolactone-synthesis gene, or of the *RwMAX2* gene. However, when a spatial light treatment was applied along the stem (*i.e.*, the three uppermost buds were placed in the dark and the three lowest buds exposed to light, causing inhibition of the uppermost buds and outgrowth of the lowest), the expression pattern of the *RwMAX1* and *RwMAX2* genes was modified. *RwMAX2* expression increased in the upper buds and internodes subjected to darkness, as reported for FR treatment [58], and this was correlated with outgrowth inhibition. Conversely, *RwMAX1* expression was promoted by light in the buds about to grow out. Such increased expression of a strigolactone-synthesis gene in an outgrowing bud is surprising but could reflect the action of a feedback loop that induces SL biosynthesis when SL signaling is repressed [144]. Overall, these results suggest that light also has an impact on strigolactone synthesis and signaling genes, and these in turn locally contribute to the control of bud outgrowth.

#### 5.3.4. Interaction with Other Hormones

A wealth of data shows that the effect of light on certain developmental processes results from the regulation of hormone-metabolizing enzymes and/or hormone-signaling pathways [147,148,149]. Expression of the GA-inactivating gene *GA2ox-2* was inversely correlated with the outgrowth potential of the buds in both *phyB* mutants and WT [150]. In *Rosa* sp., the light-induced bud outgrowth of decapitated plants went together with the control of the expression level of three GA-metabolizing genes [151]. More precisely, the inductive effect of light was associated with the rapid (within 24 h) and persistent (over 96 h) up-regulation of two GA biosynthesis enzymes (*RoGA20ox* and *RoGA3ox*) and the down-regulation of a GA-catabolizing enzyme (*RoGA2ox*). In the same vein, ancymidol and pacobutrazol, two well­known GA biosynthesis inhibitors [152,153] prevented bud outgrowth under light but their effect was completely relieved by adding exogenous GA_3_ to the medium [151]. The same synergistic effect of light and gibberellins is well documented for seed germination [154,155,156] and it differs from the effect reported during hypocotyl elongation [157]. However, no bud outgrowth was noted when rose buds were cultivated *in vitro* on GA_3_ in the dark [151]. Therefore GA-related mechanisms may be only part of an intricate mechanism behind light-induced bud outgrowth. This is in accordance with the fact that only application of CKs alone can promote SAM growth in the dark [118]. 

The involvement of ABA as a possible inhibitor of shoot branching has been considered, based on the facts that (i) increased auxin levels go along with higher ABA levels in axillary buds [158,159,160]; (ii) ABA levels in dormant axillary buds decrease in response to decapitation of the main shoot [159,161]; and (iii) exogenous ABA application inhibits bud outgrowth in *Arabidopsis*, *Ipomoea* and tomato [162,163]. A link between light and ABA in the regulation of bud outgrowth was recently reported by analyzing the mechanism behind the inhibition of axillary bud outgrowth by low R:FR in *Arabidopsis* [95,164]. The shade-mediated repression of bud outgrowth could be mediated by *BRC1*, which is necessary for maintaining ABA-related responses within buds [95]. A direct role for ABA in the repression of bud outgrowth from lower positions (in the rosette) under low R:FR was demonstrated by using the *nced3-2* and *aba2-1* ABA-biosynthesis mutants [164]. These mutants exhibited enhanced branching and defective bud n-2 outgrowth in response to low R:FR. Thus, ABA regulates bud outgrowth responses to the perception of a low R:FR, mainly transduced by PHYB [55,58,150]. *PhyB* loss of function results in increased ABA abundance in mature *Arabidopsis* leaves [165]. Hence it is tempting to speculate that the PHYB pathway reduces ABA production so that PIF or PIF-LIKE (PIL) transcription factors might activate ABA biosynthesis genes in shaded buds. Such regulation of ABA levels by light through the *Arabidopsis thaliana* gene *PIF-LIKE 5/PHYTOCHROME INTERACTING FACTOR1* (*PIL5/PIF1*) is well described for seed germination [154].

A connection between ethylene or brassinosteroid and light-mediated bud outgrowth, has not yet been demonstrated but is conceivable. Ethylene was indeed reported as a negative regulator of bud outgrowth [166,167], and in seedlings, its synthesis was shown to be inhibited by light through PHYB signaling [168]. Concerning brassinosteroids, they were shown to interact with auxin signaling and promote leaf growth [169], a developmental process of bud outgrowth and their role in light-dependent development in *Arabidopsis* was reported [149]. Further research should aim to examine their involvement in the photocontrol of bud outgrowth. 

**Figure 1:**
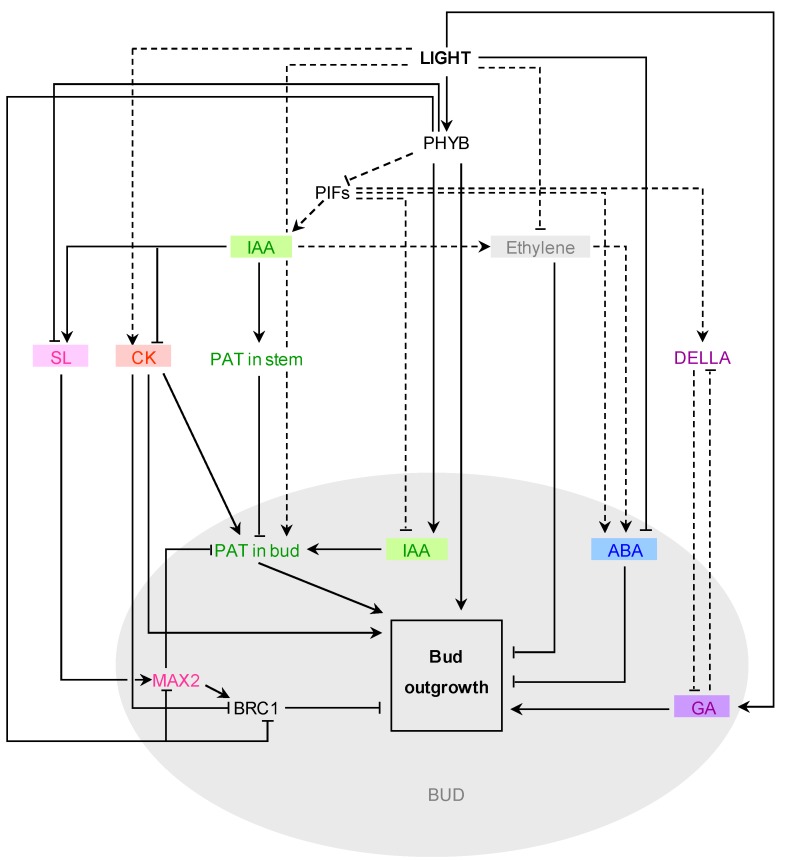
Interactions between light and hormone signaling. Full connectors represent interactions in the context of bud outgrowth, while dotted connectors represent interactions during other processes.

Figure 1 presents a working model drawn from all the results obtained so far about the interaction between these growth regulators and light in the control of bud outgrowth.

Phytochrome B (PHYB) is a major light integrator that positively regulates bud outgrowth [55]. PHYB is known to repress Phytochrome Interacting Factors (PIF) during photomorphogenesis [170]. The role of PIFs in bud outgrowth is not established yet. During seedling development, PIFs interact with auxin (IAA) synthesis and signalling [86,171] and both stimulation and inhibition of IAA synthesis by PIFs were reported [171,172]. The up-regulation of auxin synthesis genes in the bud by PHYB [55] would promote the establishment of the polarized auxin transport (PAT) between bud and stem, a prerequisite for bud outgrowth. At the shoot apex, down-regulation of auxin synthesis through PIFs regulation would rather decrease apical dominance, as apex-derived auxin inhibits cytokinin (CK) synthesis [105], promotes strigolactone (SL) synthesis [126,127] and increases PAT in the stem. PIFs could also act in the control of bud outgrowth through the control over other hormonal pathways. In seedlings, PIFs inhibit gibberellin (GA) synthesis by regulating DELLA proteins and induce abscisic acid (ABA) synthesis [154,155], two processes thought to repress bud outgrowth. Light however was shown to induce GA synthesis [151] and inhibits ABA and ethylene synthesis [164,168]. As both ABA and ethylene are bud outgrowth inhibitors, the effect of light on these two growth regulators would promote bud outgrowth. Auxin-induced ethylene production could also induce ABA synthesis [173,174] leading to bud outgrowth inhibition; such an induction could be mediated by PIF proteins [164,175]. Light, *via* PHYB, strongly affects the SL pathway by down-regulating SL synthesis, signaling (*MAX2*) and response (*BRC1*) genes [55,92]. MAX2 has a negative effect on the establishment of PAT in buds and positively controls the expression of *BRC1*. *BRC1* expression is also repressed by CKs [55,96,176], a positive bud outgrowth regulator that also promotes PAT in buds [99,129]. Light could in turn promote bud outgrowth through its positive impact on CKs signalling in SAM as shown in excised tomato apices [118].

### 5.4. Interaction of Light Signaling and Nutrients during Bud Outgrowth

Sugars regulate bud growth in many species [177,178,179,180,181]. In beheaded rose plants, buds exposed to light over-express two sugar-metabolism-related genes: (i) an *RhVI1* (*Rosa hybrida* vacuolar invertase 1) gene [182], that drives the elongation activity and sink strength of outgrowing organs [183,184,185]; and (ii) an *RhSUC2* (*Rosa hybrida* sucrose transporter 2) gene, that catalyzes the active supply of sucrose to buds [180]. In *Rosa* sp., sugars agonistically interact with light to promote the expression of *RhVI1* and bud outgrowth [186]. These findings and the fact that palatinose, a non-metabolizable sucrose analog, can mimic the effect of sucrose, suggest a cooperative effect of the light and disaccharide signaling pathways in the mediation of bud outgrowth and gene expression. A role for other sugar signaling pathways in the effect of light on bud outgrowth has also been proposed [186]. This may contribute to define the part played by well-known sugar signaling proteins such as SnrK1, Hexokinase and TOR kinase in light signaling. The development of new branches requires nitrogen (N) supply in addition to sugars; N compounds can originate from N reserve mobilization or current N uptake and assimilation [187,188]. The proportion between these two N sources during bud outgrowth is highly variable according to the species and the period of the year [189]. In beheaded one-year-old *Rosa* plants cultivated in high light conditions, N from current uptake and assimilation represented about 60% of the total N found in the new branch 4 days after bud outgrowth [190]. In addition, nitrogen deficiency strongly reduced the inductive effect of light on bud outgrowth [191]. 

### 5.5. Action of Light on Cell Growth and Expansion during Bud Outgrowth

Bud outgrowth results from the growth of bud-contained preformed leaves and their internodes and from the initiation of new ones by the meristem. During the process, growth and organogenesis involve cell division and elongation. In dormant buds, cell division is stopped and this occurs at different points of the cell cycle: in G0 in potato [192], or G1, S or G2 in pea [193], or in G1 for basal buds and G2 for upper buds along rose stems [194]. Upon bud outgrowth, the cell cycle is resumed in a synchronized manner in meristem cells. Cyclins and cyclin-dependent kinases are key proteins involved in the control of the cell cycle along with EF2 transcriptional activators and repressors [195,196,197]. Cyclin gene expression in pea is a good indicator of bud growth capacity [100,193]. In *Arabidopsis*, where cyclin D (CYCD3) stimulates cell proliferation and inhibits cell differentiation [115,198,199], *CYCD3* loss of function resulted in reduced branching and SAM size [200]. Interestingly, sugars and CKs [200,201], two main actors of bud outgrowth control, are required for CYCD3 to be active.

The initiation of new primordia at the meristem surface and the growth of preformed leaves during bud outgrowth also involve cell expansion. This process requires relaxation and then extension of the cell wall [202]. Relaxation is initiated by an increase in cell turgor, and extensibility of the wall is promoted by its hydration [203] and acidification [204,205]. For the cell wall to relax, molecular bonds between its components have to be broken, and the components have to undergo partial cleavage [206]. This is achieved by several types of proteins, among them xyloglucan-endotransglycosylases/hydrolases (XTH), endo-(1,4)-β-D-glucanases and expansins [207,208]. Upon final extension, the cell wall is strengthened by new interactions established by peroxidases and extensins [202].

Our understanding of the impact of light on cell proliferation mechanisms is currently poor. However, some reports suggest that the interaction of cell division and expansion with light may play an important role in the control of bud outgrowth.

In dormant sorghum buds, the expression of five cell-cycle genes (*SbHis4*, *SbPCNA*, *SbCycD2*, *SbCycB*, *SbCDKB*) was down-regulated by low R:FR treatment [92]. *PhyB-1* loss of function did not have the same impact as FR treatment on the expression of the cell cycle genes, suggesting that other PHYs or photoreceptors may also be involved. 

No report has been published yet on the impact of light on cell expansion during bud outgrowth. Yet, several studies on meristem functioning suggest that such regulation may takes place. For example, expansins play a key role in the induction of new initia at the meristem surface [209,210,211,212] and in the development of preformed leaves out of the apex in several species [210,213,214]. In the apex of *Arabidopsis* plants, the expression of several expansin genes was stimulated by light [215] and several *cis*-regulatory elements involved in light signaling were found in the promoter of the tomato *LeEXPA2* gene [216]. Light could also indirectly control the action of expansins through the control of intracellular pH: light activates proton pumps and thus contributes to lower intracellular pH, which in turn stimulates expansin activity [217,218].

## 6. Response of Bud Outgrowth to Light and Photosynthetic Control

Light as an energy provider and as an environmental signal deeply influences plant development through photosynthetic assimilation and photomorphogenetic responses. The question arises then as to how these two processes are involved in the control of bud outgrowth. From a trophic viewpoint, this question is of great importance because an outgrowing bud is a strong sink organ that actively imports the sugars required for meeting its high metabolic demand [177,181].

As described above, increased branching was observed in several species under increasing light intensities. For some authors, such increased branching is part of an overall strategy of the plant aimed at allocating its resources to organs that better catch light and give the plant a better carbon return than the promoted growth of existing branches [20,219]. For example, in the shrub *Vaccinium hirtum*, increased light availability stimulated the production of new sprouts from the base of the plants but did not promote the growth of existing stems [219]. In *Picea abies*, more sylleptic shoots were formed at the top of the crown under higher irradiance, leading to a larger number of branches and branch concentration in the upper canopy where large amounts of photons can be harvested [19].

In these studies, the signal that triggers branching is expected to work at the whole plant level through altered resource allocation towards certain buds rather than through a direct impact of light on photosynthetic activity at the branching site. Still, since buds contain green tissues (green scales, young leaves, primordia and SAMs), we can wonder whether the impact of light on bud outgrowth is under photosynthetic control at the branching site. In other words, we do not know if bud exposure to low light intensity or to lower quantum efficiency light qualities such as blue light [220,221] reduces photosynthesis in bud organs and impairs their capacity to grow out. This question has not been directly addressed yet and will require future investigations. 

Excised tomato shoot apices cultured *in vitro* provided a few answers about the interaction between photosynthesis and meristem organogenic activity: under light, new organs were produced by tomato SAMs on a sugar-supplemented medium [118]. When these same shoot apices were exposed to darkness, SAM activity was totally inhibited and no new organs were produced, evidencing a photocontrol of SAM activity in tomato. This response to light was not due to the regulation of the photosynthetic activity of tomato apices: when Norflurazon, a photosynthesis inhibitor, was added to the medium under light treatment, although tissues bleached due to the inhibition of photosynthetic pigment synthesis, SAM organogenic activity resumed with the same efficiency [118]. In *Rosa*, the growth of young shoots, (2–3 cm produced six days after plant pruning under light) was deeply affected by shade or darkness that caused their terminal flower bud to abort [222]. When these shoots were sprayed with DCMU, another photosynthesis inhibitor, the promoting effect of light on shoot development and organogenesis appeared independent of their photosynthetic assimilation [222]. In *P. dilatatum*, reduced tillering due to low R:FR was fully reversed by applying small, photosynthetically negligible amounts of R to the base of the plants. This illustrates the major role of photomorphogenesis processes over photosynthesis at the site of branching in the control of bud outgrowth [38].

Still, in order for plants to best adapt to a changing environment, photosynthetic activity and photomorphogenic processes are bound to interact to modulate plant development and branching. Recent works show such an interaction in *Arabidopsis thaliana* [150]. In this species, mutation of *PHYB*, the main photoreceptor involved in the sensing of R:FR, caused reduced branching compared to the WT. However, such reduced branching only occurred under low PPFD (160 µmol∙m^−2^∙s^−1^). When light intensity increased to 280 µmol∙m^−2^∙s^−1^, the branching level in *phyB* mutants reached the same level as in WT plants. This increased branching is due to a greater capacity of buds to grow out, not to higher numbers of leaves and buds produced by the plants under higher light intensity*.* This result demonstrates that (i) the suppressive effect of *phyB* mutation on plant branching can be overridden by higher light intensity; and (ii) there are interactions between photosynthesis and photomorphogenesis in the control of bud outgrowth. The expression of genes involved in bud outgrowth control led the authors to suggest that (i) PPFD and PHYB act both cooperatively and independently to regulate branching; and (ii) light, through PPFD, could influence branching through a specific signaling pathway. This is thought to allow plants to best respond to changing light environments, for example by allowing branching when R:FR is reduced by not-yet shading neighboring plants under high PPFD. 

If photosynthesis interacts with photomorphogenesis in the control of bud outgrowth, which are then the mechanisms involved in this interaction? Several pigments (chlorophyll, carotenoids, anthocyanins, *etc.*) collect light for photosynthesis, and their amounts can be modulated by plants according to light conditions [147]. Several reports show that photoreceptors are involved in the regulation of photosynthetic pigment content. This illustrates the interconnection between photomorphogenesis and photosynthesis. For example, in tomato, the *cry1* mutation reduces chlorophyll content in leaves [62], while overexpression of *CRY2* has the reverse effect [64]. In *Arabidopsis*, a complex interaction between PHYA, PHYB and CRY1 is involved in the control of the chlorophyll content of seedlings; the triple *phyAphyBcry1* mutation dramatically decreased chlorophyll levels in the young plants [223]. Can the regulation of photosynthetic pigment content in the bud itself, or in the nearby green organs, be a mechanism involved in the control of bud outgrowth? Or does such a regulation act only at the whole plant level, impair plant photosynthetic activity, and act on resource allocation towards buds as discussed above? Such issues would be interesting to address. 

## 7. Conclusions

Although the impact of light on bud outgrowth is well established, this review shows that we know little about how light controls this process. A wealth of information can be gathered from other earlier studied developmental processes (seed germination, seedling development, flowering), but the specific actors of the transduction of light signals in the control of bud outgrowth remain to be unveiled. Challenging questions address the autonomous *versus* non-autonomous nature of bud response to light, and the part played by inter-organ signaling in this response. Not much is known either on the part played by the different photoreceptors, their redundant effects and their major molecular partners in the control of bud outgrowth. This challenging research work will contribute to a better understanding of how a plant builds up its architecture, but should also offer new tools to master crop compactness and yield and increase phytosanitary protection.
